# SARS-CoV-2 Ct values and COVID-19 symptoms in patients with haematological malignancies in South Africa

**DOI:** 10.4102/sajid.40i1.676

**Published:** 2025-01-09

**Authors:** Keitumetsi L. Sothoane, Sarah A. van Blydenstein, Vinitha Philip, Jeannette Wadula

**Affiliations:** 1 Department of Internal Medicine, Faculty of Health Sciences, University of the Witwatersrand, Johannesburg, South Africa; 2 Division of Pulmonology, Department of Internal Medicine, Chris Hani Baragwanath Academic Hospital, Faculty of Health Sciences, University of the Witwatersrand, Johannesburg, South Africa; 3 Division of Clinical Haematology, Department of Internal Medicine, Chris Hani Baragwanath Academic Hospital, Faculty of Health Sciences, University of the Witwatersrand, Johannesburg, South Africa; 4 Department of Clinical Microbiology & Infectious Diseases, Chris Hani Baragwanath Academic Hospital, Faculty of Health Sciences, University of the Witwatersrand, Johannesburg, South Africa; 5 National Health Laboratory Service, University of the Witwatersrand, Johannesburg, South Africa

**Keywords:** severe acute respiratory syndrome coronavirus 2 (SARS-CoV-2), COVID-19, cycle threshold value, haematological malignancies, symptoms, viral shedding, chemotherapy

## Abstract

**Background:**

Severe acute respiratory syndrome coronavirus 2 (SARS-CoV-2) reverse transcriptase polymerase chain reaction (RT-PCR) cycle threshold (Ct) values serve as surrogate markers for estimating viral load. Their usefulness in patients with haematological malignancies and COVID-19 has not been studied in the South African context.

**Objectives:**

To evaluate if a Ct value < 30 can predict COVID-19 symptom development in adult patients with haematological malignancies.

**Method:**

A retrospective cohort study on adult patients with haematological malignancies and COVID-19 was conducted at Chris Hani Baragwanath Academic Hospital from 01 July 2020 to 31 July 2021. The relationship between Ct values, symptoms and disease severity, along with changes over time were evaluated.

**Results:**

Among 53 patients (50.9% male, median age of 38 years), Ct values < 30 did not significantly predict COVID-19 symptom development (*p* = 0.417). However, severe disease correlated with lower Ct values (*p* = 0.002). No significant difference in the duration (days) from positive to negative tests was found between symptomatic and asymptomatic patients, and by severity of disease in the symptomatic patients. Lymphopenia was associated with severe disease, and those with lymphoid malignancies experienced longer viral shedding.

**Conclusion:**

Patients with haematological malignancies can exhibit symptoms at any Ct value but lower Ct values indicate more severe disease. This information can be critical for chemotherapy timing to minimize adverse outcomes.

**Contribution:**

The findings suggest a potential benefit in delaying chemotherapy at any Ct value as patients could present with acute SARS-CoV-2 infection at higher Ct values, and therefore face increased risk of adverse outcomes with early chemotherapy initiation.

## Introduction

The clinical manifestation of infection with Severe acute respiratory syndrome coronavirus 2 (SARS-CoV-2) ranges from mild self-limiting disease to life-threatening disease which presents as systemic inflammation with multiple organ involvement.^1^

The gold standard for the laboratory diagnosis of SARS-CoV-2 involves a nucleic acid amplification test using a specimen obtained via a nasopharyngeal (NP) or oropharyngeal (OP) swab.^2^ This virus-specific (semi-) quantitative reverse transcriptase polymerase chain reaction (RT-PCR) assay replicates the viral ribonucleic acid (RNA) from the patient’s sample until it has a detectable concentration that exceeds the set threshold value. The number of cycles necessary for that to take place is known as the Cycle threshold (Ct) value.^3^ Thus, Ct values correlate with viral loads – the lower the Ct value of a patient’s sample, the higher the viral load. The use of SARS-CoV-2 RT-PCR Ct values as simple markers of new or active infection and for disease severity allow for easy identification and triaging of high-risk cases and infectivity.

In patients hospitalised with SARS-CoV-2 infection, those with lower Ct values had more severe disease necessitating non-invasive ventilation, mechanical ventilation or Intensive Care Unit (ICU) admission. Higher mean Ct values were seen among survivors compared with non-survivors. This association was most notable when comparing patients with Ct values < 25 to those with Ct values > 30.^3^

Although one might assume that the likelihood of infectivity decreases as the Ct value increases,^4^ RT-PCR does not evaluate the infectious capacity of the detected RNA. No live virus was isolated from sample cultures obtained 8 days after the onset of symptoms.^2^ The probability of infection is dependent on exposure time, the infectious dose and viral shedding. Infectious SARS-CoV-2 virus was detected in clinical samples even at low concentrations of viral RNA, as indicated by a Ct value of 33. Cycle threshold values ranging from 17 to 32 were associated with culturable amounts of the virus and were therefore presumed to indicate infectious potential.^5^ At Ct values > 30, it was possible to successfully culture the virus; although, the likelihood of successful culturing decreased to 6%.^4^ A culturable virus may be shed more than 10 days after onset of symptoms, in spite of a Ct value > 35.^5^

In previously infected cells, RNA viral loads gradually declined over the course of the disease reaching low or undetectable levels 2 weeks after the onset of symptoms. Declining RNA viral load is associated with resolution of clinical symptoms and gradual increase in antibody titres, for both binding and neutralising antibodies.^2^

The persistence of viral RNA may not necessarily be associated with disease severity but may indicate that the immune response is unable to promote the virus RNA clearance as shown in patients who tested positive for more than 2 weeks.^6^ Immunosuppression has been documented to delay the clearance of the SARS-CoV-2 virus.

In a 2021 South African study, among those with high initial SARS-CoV-2 viral load (Ct < 30), the duration of viral shedding was 8 days overall and significantly prolonged in people living with HIV (PLHIV), particularly with HIV CD4 counts < 200 cells/µL and/or high HIV viral load.^6^ Overall, lower median anti-spike protein antibody titres were detected in PLHIV. Over 70% of the individuals, who were PLHIV, did not develop antibodies within the initial 21 days follow-up period, and almost half of whom showed prolonged viral shedding.^7^

A 2020 retrospective observational study conducted in King’s College Hospital evaluated the outcomes and predictive factors in haematology-oncology patients hospitalised with coronavirus disease 2019 (COVID-19), compared to patients without malignancies. They found a two-fold increased risk of mortality, with a 28-day mortality rate of 39%. This rate was four times higher in patients currently undergoing treatment for their malignancy.^8^ The type of underlying malignancy did not influence the outcome. Worsening of lymphopenia during the infection and the depth of lymphopenia prior to infection had an impact on survival. This is in line with several recent studies suggesting that over- and/or under-activation of the adaptive immune system may be responsible for the high mortality associated with COVID-19.^8^ The median duration of virus detection in the respiratory samples was 29 days in 80 patients with haematological malignancies. Only a small percentage became negative at a median of 13 days (range 7–60) and the majority (59%) had RNA persistence at 13 days.^8^

Persistent symptomatic COVID-19 was seen in patients with lymphoid malignancies, a condition worsened by previous anti-CD20 therapy and observed in 21% of anti-CD20 treated patients.^8^ Immune dysregulation, which is characterised by combined loss of B-cell function and impaired CD4^+^ T-cell counts and compensatory innate immunity and CD8^+^ T-cell activation, was said to be the basis for this chronic syndrome. The syndrome manifests as a chronic protracted illness marked by slowly progressive or relapsing respiratory illness with persistent viral RNA detection.^8^

Prolonged persistence in haematology-oncology patients has significant implications for scheduling subsequent chemotherapies and self-isolation. To our knowledge, there are currently no studies in Africa that evaluate the value and correlation of Ct value with COVID-19 disease symptom presentation, severity and viral shedding in patients with haematological malignancies. We therefore aim to investigate the association between the presence of symptoms and Ct values < 30, with the goal of identifying if this Ct value may be statistically significant in indicating active disease. This determination could aid in decision-making regarding the continuation or postponement of chemotherapy.

To date, only 40.2% of South Africans completed the primary COVID-19 vaccination programme (fully vaccinated, excluding booster doses).^9^ In a 2024 study looking at data from 2020 to 2022 from the EPICOVIDEHA registry, 64.5% (*n* = 5658) of patients with haematological malignancies who were previously diagnosed with COVID-19 before the availability of vaccines were still unvaccinated, and exhibited the highest mortality rate at 20.8%.^10^ Currently, there is no data on the number of patients with haematological malignancies who are fully vaccinated for COVID-19. Despite previous COVID-19 infection in some patients, those with haematological malignancies are less likely to mount an immune response and sufficient immunoglobulin titres, leading to higher rates of reinfection. Therefore, this makes our study applicable and relevant to date.

## Research methods and design

### Study design

We conducted a retrospective cohort study from 01 July 2020 through 31 July 2021, during the pre-vaccination era, spanning the first two waves of the COVID-19 pandemic in South Africa (ancestral virus and beta variant virus, respectively).^11^

### Study setting

Chris Hani Baragwanath Academic Hospital (CHBAH) is a South African tertiary hospital in the South-Western Township of Johannesburg. The Clinical Haematology Department manages both benign and malignant haematological conditions, and receives new patients from within the hospital and as referrals from other institutions.

### Study population

#### Inclusion criteria

Males and females 18 years and older with a diagnosis of a haematological malignancy (lymphoid, myeloid or plasma cell dyscrasia); patients requiring inpatient management of their haematological malignancy; a laboratory confirmed diagnosis of SARS-CoV-2 infection on admission OR a hospitalised individual who developed symptoms suggestive of SARS-CoV-2 infection or disease after 48 h of their admission; and patients who tested positive for the first time for SARS-CoV-2 in the institution (no previously documented SARS-CoV-2 infection or disease in the institution prior to the study dates).

#### Exclusion criteria

Patients younger than 18 years of age; patients not requiring inpatient management that is outpatient chemotherapy; a negative or inconclusive SARS-CoV-2 RT-PCR laboratory diagnosis; patients with previously documented SARS-CoV-2 infection or disease in the institution prior to the study and in their recovery phase at the beginning of the study; and patients with files not retrievable.

### Sample size

**FIGURE 1:**
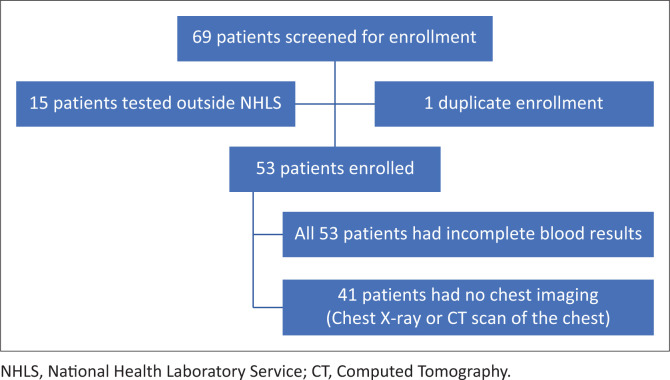
Schematic representation of patients enrolled in the study with retrievable cycle threshold values.

We included 53 patients in the study. See Figure 1 for the selection process.

### Data collection procedure

Data from the Clinical Haematology Department was used to screen for candidates who met the inclusion criteria for the period under investigation. In addition, inpatients who tested positive were flagged by the National Health Laboratory Service (NHLS) to the department, and were included in the study.

A laboratory diagnosis of SARS-CoV-2 was determined as follows:

- A NP swab specimen was collected by the admitting doctor in the Medical Emergency Unit (MEU), Medical Admissions Ward or Clinical Haematology Outpatient Department (HOPD) on patients admitted for the management of a haematological malignancy. This was according to the CHBAH COVID-19 standard operating procedure (SOP) requiring all patients for admission to be tested for SARS-CoV-2; with or without symptoms. If the individual was already in the hospital for treatment and had developed COVID-19 symptoms or disease during their hospital admission, a NP swab collected by the medical doctor in the respective medical ward was utilised.
- The NP swabs were collected using flocked nylon plastic swabs which were inserted into the universal transport medium and transported on ice to the laboratory.
- Reverse transcriptase polymerase chain reaction for the qualitative detection (extraction and amplification) of nucleic acid for SARS-CoV-2 was performed on NP specimens using either TaqPath RT-PCR COVID-19 Kit (Thermo Fisher Scientific) *QuantStudio*™ 5 Dx Real-Time PCR Instrument, GeneXpert (GXP) OR Allplex Assay (Allplex 2019-nCOV Assay) Seegene-C*FX96* Touch Real-Time PCR instrument as provided by the on-site NHLS laboratory.
- Specimens were considered positive for SARS-CoV-2 if at least two of the three genes were positive (Ct value < 40 for SARS-CoV-2 N gene, ORF1ab gene or S gene when using the TaqPath^TM^ COVID-19 MS2 Combo Kit 2.0 multiplexed real-time PCR test or SARS-CoV-2 E gene, RdRP gene or N gene when using Allplex Assay).
- In the case of GXP, specimens were considered positive for SARS-CoV-2 if both the N2 and E gene targets had Ct value < 40.^12^ If a single gene exhibited a Ct value > 38, the result was classified as a presumed positive or inconclusive, and thus did not fulfil the criteria for a definitive positive test (therefore excluded from study).
- Cycle threshold values for each successful amplification of a gene were obtained using the above-mentioned analysing systems, for every positive SARS-CoV-2 PCR result.
- A mean cut-off Ct value of < 40 derived from the three genes on NP specimens was used as a proxy for probable acute infection with COVID-19 as per national NHLS testing guidelines. This cut-off was based on the Ct ranges established by each assay manufacturer, which ranged between 5 and 43 when a specimen is reported positive for the detection of SARS-CoV-2.^12^
- The internal NHLS result database at the investigation site was used to ascertain which RT-PCR amplifying and analysing system was used for each individual result, along with the corresponding Ct values.

Nasopharyngeal swabs were repeated at 2 weekly intervals from the initial positive test (after 14 days of self-isolation) in patients who were being admitted from HOPD for their scheduled inpatient chemotherapy. Chemotherapy was delayed until a negative NP swab was achieved.

Patients who contracted COVID-19 as inpatients following admission for inpatient chemotherapy were isolated in the COVID-19 Ward until discharge. Their repeat NP swabs were repeated when they required subsequent admission for their next scheduled inpatient chemotherapy. In this instance, the time interval to their next NP swab was patient-tailored and therefore resulted in a lack of standardisation for subsequent tests in relation to all patients.

### Definitions

- *Asymptomatic or Presymptomatic Infection or Coincidental*: Individuals who test positive for SARS-CoV-2 using a virologic test (a nucleic acid amplification test) but who have no symptoms that are consistent with COVID-19.^13^
- *Mild illness*: Individuals with general symptoms^3^ and a Room Air Oxygen Saturation greater than 93% at rest.^14^
- *Moderate Illness*: In addition to general symptoms, individual who have a room air oxygen saturation < 94% requiring supplemental oxygen support with either nasal prong cannula or face oxygen mask.^13^
- *Severe Illness*: In addition to general symptoms, individuals having a Room Air Oxygen Saturation less than 94%, a ratio of arterial partial pressure of oxygen to fraction of inspired oxygen (PaO_2_/FiO_2_) of less than 300 mm Hg, a respiratory rate greater than 30 breaths/min or lung infiltrates greater than 50%,^13^ multiple organ dysfunction, with deranged biochemical markers or who require organ support in addition to invasive ventilatory support.^3,13,14,15^
- *Positive SARS-CoV-2 test*: Presence of SARS-CoV-2 nucleic acid in a specimen as detected by a positive SARS-CoV-2 RT-PCR result from an OP or NP swab, if the Ct value was < 40 for ≥ 2 of gene targets.^13,14^
- *Viral shedding*: Persistent presence of SARS-CoV-2 nucleic acid in a specimen as detected by a positive SARS-CoV-2 RT-PCR result. Individuals were deemed to have stopped shedding SARS-CoV-2 from the respiratory tract once a swab tested negative for all gene targets on RT-PCR. Time to cessation of shedding was taken from date of symptom onset to the date of the last RT-PCR SARS-CoV-2 positive NP swab prior to the consecutive negative swab.^13^

### Data management

- Data extrapolated from the patients’ records included blood results from the internal NHLS database at the investigation site and NHLS LABTRAK/TrakCare website or application, vital signs and oxygen requirements, and imaging studies retrieved from the CHBAH Picture Archiving And Communication System (PACS).
- REDCap Database was designed for the study, and data was captured by the first author.
- Personal identifying information was replaced with research identification codes. Each patient was assigned a randomised double-digit number using an online random number generator.
- If third parties require sharing of data, necessary applications to the HREC and NHLS will need to be conducted accordingly and await approval.

### Statistical analysis

The study data captured in the REDCap database^16,17^ was downloaded as a Stata version 18.0 (Stata Corporation, College Station, US file) file. Following data cleaning, which included removing duplicates and checking for inconsistencies, descriptive analysis was conducted to describe the patients included in the study. Frequencies and percentages were used to describe the patients with respect to categorical variables. Medians and interquartile ranges (IQRs) were used for continuous variables with means and standard deviations used for normally distributed continuous variables.

#### Primary objective

Cycle threshold values were the main exposure variable and continuous variable, ranging from 0 to 40. For each patient, a mean Ct value was calculated using the three Ct values obtained from the three target genes. In cases where GXP was used, the mean Ct value was derived from Ct values of the two target genes. From these mean Ct values, we were able to derive mean and median Ct values for the study population. The median Ct values were compared between symptomatic and asymptomatic sub-groups using the Wilcoxon rank-sum test to test the hypothesis. Receiver-operator-curve (ROC) analyses were used to determine the sensitivity and specificity of the average Ct values in predicting the presence of symptoms. Overall, Ct value performance was determined from the area under the curve (AUC) with a 95% confidence interval (CI). The selected Ct value was the one that best predicted between symptomatic and asymptomatic patients. Other exposure variables were age, biological sex and underlying haematological malignancy.

#### Secondary objectives

Descriptive analysis was used to determine the median of the mean Ct values for the population and presented as IQRs by presence of symptoms and severity of disease. The Wilcoxon rank-sum test was used to test the hypothesis that the median Ct values were different between patients who developed symptoms and those who did not, as well as those with severe disease compared to those without severe disease. The time from testing positive to negative was determined as time in days from the first positive test to the first negative test, and summarised as a median with an IQR. The Wilcoxon rank-sum test was also used to test the hypothesis that the median time from positive to negative was different between patients who developed symptoms and those who did not. The Pearson correlation coefficient with a 95% CI was used to determine the correlation between mean Ct values and time from positive to negative test. The same hypothesis was used when illustrating changes in time of the mean Ct value during the illness.

### Ethical considerations

Ethical approval to conduct this study was obtained from the University of the Witwatersrand Human Research Ethics Committee (Ref no: M220816 MED22-08-038), Johannesburg, South Africa, and the National Health Research Database (M220816). A consent waiver from ethics was granted under the circumstances that the study was a review of information in the public domain, and that there are no active human participants.

## Results

A total of 69 patients were assessed for eligibility. From whom, 15 patients were excluded on the basis of having their SARS-CoV-2 RT-PCR NP specimens processed outside the NHLS, and therefore had no retrievable Ct values. One participant was a duplicate entry. Around 77.3% of the patients had no retrievable chest imaging; therefore, chest imaging findings were not included in the study (Figure 1).

Of the remaining 53 patients, 14 (26.6%) had myeloid malignancies, 35 (66%) had lymphoid malignancies and 4 (7.4%) had plasma cell dyscrasias which included multiple myeloma. The median age at presentation was 38 years (IQR 28–47 years), and just over half of the patients were male (50.9%, *n* = 27) (Table 1). 43% of the tests were conducted using TaqPath RT-PCR COVID-19 Kit (Thermo Fisher Scientific) *QuantStudio*™ 5 Dx Real-Time PCR Instrument, 25% using GXP, 26% using Allplex Assay (Allplex 2019-nCOV Assay) Seegene-C*FX96* Touch Real-Time PCR and 6% using Cobas SARS-CoV-2 Roche Molecular Assay. Over half (58.5%) of the patients who tested positive for SARS-CoV-2 were asymptomatic. Most of the patients, including those with symptoms, were not oxygen-requiring (84.9%). Of those with COVID-19 disease, 88.6% had lymphopenia (0.99 [0.42–1.47], *n* = 47] and 66% had an elevated C-reactive protein (137 [38–266], *n* = 35) (Table 1).

**TABLE 1:** Demographic and clinical characteristics of patients with haematological malignancies who tested positive for SARS-CoV-2 at or during admission (*N* = 53).

Variables (Patients = *n*)	*n*	%	Median	IQR
Age (Years)	53	100.0	38.00	28–47
Sex (Male)	27	50.9	-	-
**Haematological Malignancies**	53	100.0		
Myeloid Malignancies	1	26.6	-	-
Lymphoid Malignancies	35	66.0	-	-
Plasma Cell Dyscrasias	4	7.4	-	-
**Symptoms**	53	100.0		
No	31	58.5	-	-
Yes	22	41.5	-	-
**Required oxygen**	53	-	-	-
No	45	84.9	-	-
Yes	8	15.1	-	-
**Symptom score severity**	22	100.0		
Mild	13	59.1	-	-
Moderate	4	18.2	-	-
Severe	5	22.7	-	-
WCC	50	-	5.01	3.16–9.22
ANC	48	-	3.06	0.94–6.00
ALC	47	-	0.99	0.42–1.47
Platelets	50	-	179.00	51–323
CRP	35	-	137.00	38–266
LDH	17	-	725.00	415–1620
AST	28	-	32.00	18.5–40
ALT	28	-	23.50	12–41
Ferritin	14	-	1149.00	364–1533
D-Dimer	18	-	1.46	0.64–2.57
Troponins	7	-	18.00	10–85
PCT	6	-	0.49	0.13–0.81

Note: Data expressed as median and interquartile range (IQR) or percentage count *n* (%).

WCC, White cell count; ANC, Absolute neutrophil count; ALC Absolute lymphocyte count; CRP, C-reactive protein; LDH, Lactate dehydrogenase; AST, Aspartate transaminase; ALT, Alanine transaminase; PCT, Procalcitonin.

### Cycle threshold values and presence of coronavirus disease 2019 disease symptoms

**FIGURE 2:**
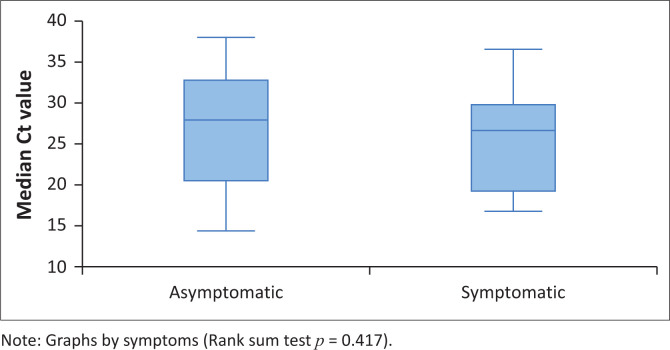
Distribution of cycle threshold values by presence of symptoms among patients with haematological malignancies who tested positive for SARS-CoV-2 at or during admission (*N* = 53).

The median Ct value for patients who presented with COVID-19 disease symptoms was 26.6 (IQR 19.9–29.2), while the median Ct value for patients who were asymptomatic was 27.8 (IQR 20.5–32.7) (Figure 2). There was no statistical significance in Ct values < 30 and COVID-19 disease symptom development (*p* = 0.417); therefore, having symptoms was not associated with Ct values in patients with haematological malignancies.

**FIGURE 3:**
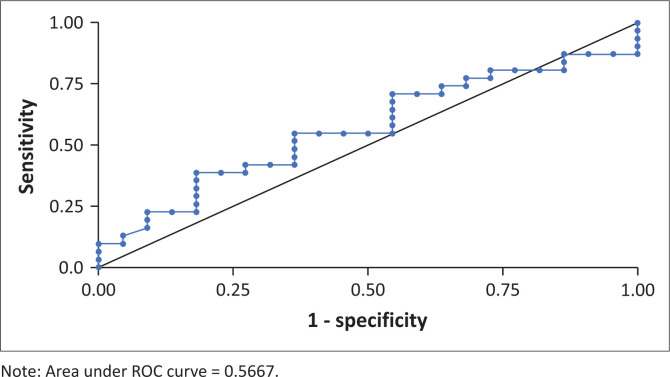
Performance of median cycle threshold values in predicting symptoms among patients with haematological malignancies who tested positive for SARS-CoV-2 at or during admission (*N* = 53).

The AUC in the ROC curve demonstrated that Ct values were not a good predictor of presence or absence of symptoms, and there was no single average Ct value that best predicted the presence or absence of symptoms in patients with haematological malignancies (AUC = 0.57 [95% CI 0.41–0.72]) (Figure 3).

### Cycle threshold values and severity of disease

**FIGURE 4:**
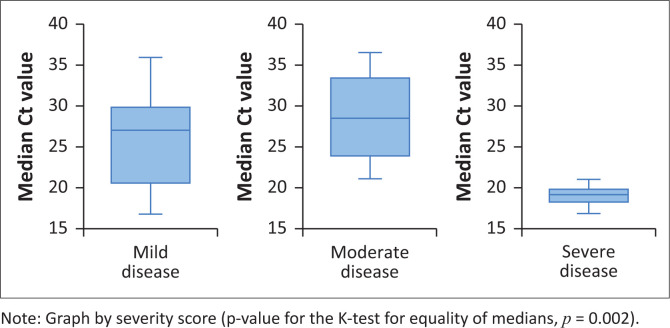
Distribution of cycle threshold values by severity of disease among symptomatic patients with haematological malignancies who tested positive for SARS-CoV-2 at or during admission (*N* = 22).

The grading of disease severity was established using retrievable data on symptoms reported on presentation, oxygen requirements and blood results. The cohort with mild disease had a median Ct value of 26.5 (IQR: 18.0–29.6); those with moderate disease had a median Ct value of 26.6 (IQR: 21.1–31.9); and those with severe disease had a median Ct value of 19.5 (IQR: 18.2–21.1). There was a statistically significant association between Ct values and severity of disease in patients with haematological malignancies (*p*-value for the K-test for equality of medians = 0.002). The more severe the disease, the lower the Ct values, and therefore the higher the viral load or viral burden (Figure 4).

### Time from positive test to negative test

A total of 21 patients in the cohort had no retrievable or repeat SARS-CoV-2 RT PCR NP swabs done; therefore, only 32 patients were evaluable. The median time taken from positive to negative test on repeated NP swabs was 43.5 days (IQR: 20.5–102 days).

**FIGURE 5:**
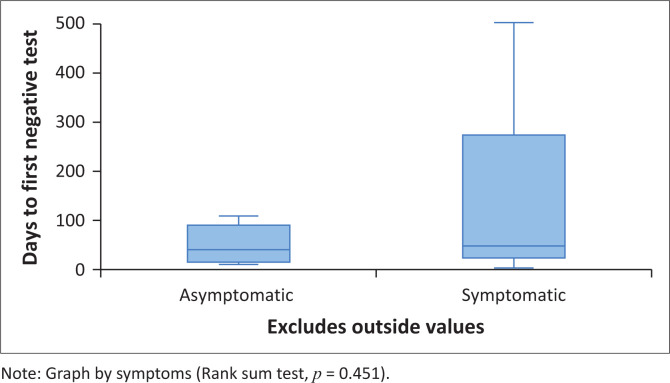
Distribution of time to negative results by symptoms among patients with haematological malignancies who tested positive for SARS-CoV-2 at or during admission (*N* = 32).

For symptomatic patients, the median time taken from positive to negative test was 49 days (IQR: 23.5–277 days) *n* = 16; and for asymptotic patients, the median time taken was 41.5 (IQR: 14.5–91.5 days) *n* = 16. There was no statistically significant difference in the median number of days taken from positive to negative tests between patients who had presented with symptoms and those who were asymptomatic (rank sum test *p* = 0.451). However, the number of days to negative test was skewed to the right, with a few patients having very long intervals between negative to positive tests due to multiple reasons (Figure 5).

**FIGURE 6:**
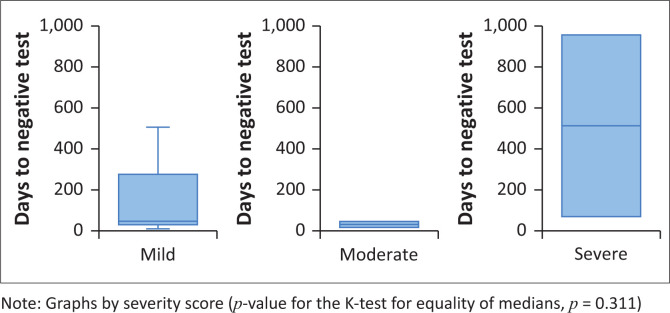
Distribution of time to negative results by disease severity among symptomatic patients with haematological malignancies who tested positive for SARS-CoV-2 at or during admission (*N* = 16).

The time taken from positive to negative was further evaluated in patients who had symptoms, and comparisons were made based on the severity of the disease. Patients presenting with mild symptoms had a median of 48 days (IQR: 24–308 days) to a negative test, 50 days (IQR: 45–89 days) for patients with moderate symptoms, and 511.5 days (IQR: 58–965 days) for patients with severe disease (Figure 6). There was no statistically significant difference in the median time from positive to negative by severity of disease among those who had symptoms (*p*-value for the K-test for equality of medians, *p* = 0.311). The interpretation of these results was severely limited by the number of patients for which time to negative test was available.

### Cycle threshold value and changes in time during illness

**FIGURE 7:**
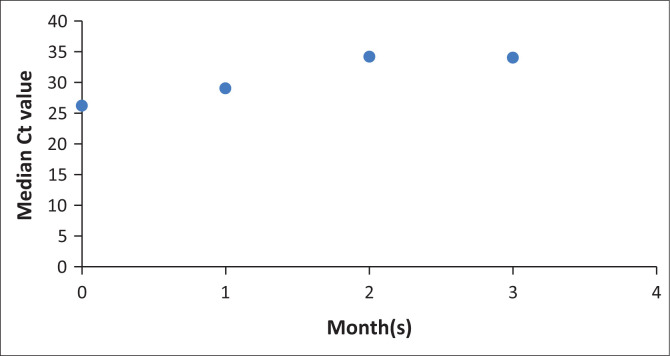
Evolution of median cycle threshold values over time among patients with haematological malignancies who tested positive for SARS-CoV-2 at or during admission (*N* = 32).

The median Ct value at baseline for the entire cohort was 26.4 (*n* = 53). The change in Ct value was evaluated for every repeated positive COVID-19 test, and the changes were represented using values from each month. At the first month following the initial positive test, the median of the patients’ mean Ct value was unchanged at 29.2 (*n* = 7), at month two it was 34.4 (*n* = 4); and by month 3 it was 34.2 (*n* = 2). After the 3rd month, there was one NP swab which was evaluable (Figure 7).

## Discussion

The main findings of our study are that patients with haematological malignancies may or may not present with COVID-19 disease symptoms at Ct value < 30; a lower Ct value and lymphopenia are associated with severe disease; patients with lymphoid malignancies have prolonged viral shedding; and irrespective of symptom presence or absence, Ct values had no predictable change in value over time.

The two SARS-CoV-2 RT PCR assays used, The Allplex™ SARS-CoV-2 assay and The TaqPath™ COVID-19 assay, were the first tests to be implemented by NHLS in March and May of 2020, respectively. The former contributed a national test count of 26% by the NHLS and the latter 21%.^12^ In 2020, an observational study was conducted to compare and evaluate seven commercially available kits for diagnostic accuracy. Both the TaqPath Kit and Allplex Assay detected all sample pools with low *Ct* values (< 30) while testing weak positive pooled samples with high *Ct* value (> 30), and showed 100% sensitivity, specificity, and accuracy.^18^ The Ct values for these assays were also found to be affected by variants of concern, especially in the B.1.617.2 (Delta) variant for the Allplex assay and the B.1.1.7 (Alpha) and the B.1.1.529 (Omicron) in the TaqPath assay.^12^ This, however, did not apply in our study as our research was conducted during the first two waves (ancestral virus and beta variant virus, respectively).

Two-thirds of our patients who tested positive for SARS-CoV-2 had a lymphoid malignancy. Lymphoid malignancies increased the risk of COVID-19 infection and adverse outcome.^19^ This is because of reduced pre-existing SARS-CoV-2 cross-reactive CD4^+^ T-cell response and T-cell exhaustion compared to patients with solid malignancies and healthy volunteers.^20^ In B-cell malignancies, there’s impaired innate humoral response especially in individuals who have received anti-CD20+ monoclonal antibodies. Lower antibody levels against SARS-CoV-2 spike protein is thought to cause delayed neutralising antibody release.^1^ Therapy for B-cell malignancies may also impair specific T-cell response antiviral activity. Patients with a greater number of CD8+ T cells have an improved survival, regardless of previous anti-CD20+ therapy.^1^

A total of 88.6% of our patients had lymphopenia while 66% had an elevated C-reactive protein. There is a significant association between lymphopenia and disease severity,^21^ and the worst outcome is associated with C-reactive protein > 10 mg/dL at admission.^21^ The lymphocyte count is a simple, effective and cheap guide to predict disease severity and mortality outcome among COVID-19 patients. This is especially important in low-and middle-income countries, where a lack of resources may limit expensive investigations. Earlier detection of lymphopenia should alert physicians to monitor for deterioration and investigate appropriately.^21^

We found that the established Ct value does not predict COVID-19 disease symptom development in adult patients with haematological malignancies. This finding was similar to a 2022 study that included 202 patients who were hospitalised with COVID-19 and had other underlying co-morbidities excluding haematological malignancies.^22^ However, another study found that 44% of symptomatic patients (without haematological malignancies) had a low Ct value (Ct ≤ 24).^23^

In a prospective study from Boston University in Massachusetts, involving a cohort of 1633 students and staff members without haematological malignancies, trends revealed that asymptomatic individuals exhibited the highest Ct values. Symptomatic individuals had the lowest Ct values. For primer-specific viral antigens, although not specified, the nucleocapsid antigens 1 (N1) and 2 (N2) were measured, with median Ct values of 21.8 (IQR 17.2–29.4) for N1 and 21.4 (IQR 17.3–28.9) for N2.^24^ In a separate case series involving 15 patients with haematological malignancies, Ct values at diagnosis were lower in patients who eventually developed COVID-19 pneumonia (9 out of the 15 patients).^25^

Patients with haematological malignancies may or may not present with symptoms at any given Ct value, and this is largely dependent on the host’s ability to mount an innate immune response in the early stage of the infection, which can be affected by various factors. These are mainly age, co-morbidities and active treatment received. If the host is able to mount a strong interferon-mediated response (Type I IF mediated by CD4+ T cells) at early stages of the infection, they may present with symptoms, control viral replication and limit disease severity at this stage through increased viral clearance. Patients with all types of haematological malignancies have T-cell defects. Therefore, one might postulate that one needs a functional innate immune system to present with symptoms.

We found that there was statistical significance between Ct values and the severity of disease in patients with haematological malignancies. This finding was similar to a 2022 study from Spain. The highest mortality rate in their cohort was observed among patients with a short duration of symptoms onset to severity, and a Ct value ≤ 25.^26^ In patients hospitalised with COVID-19, those with lower CT values had more severe disease necessitating non-invasive ventilation.^8^ In another case series, Ct values were lowest in severe forms of COVID-19.^25^

In a 2023 study from Qatar, there was no association found between Ct value and COVID-19 criticality and mortality, and that respiratory and gastrointestinal symptoms were strongly associated with higher odds of ICU admission and mortality.^27^ Abdulrahman et al. also found no significant correlation between oxygen requirements and respiratory indices on admission (eluding to severe disease) and Ct value.^26^

We found no statistically significant difference in the median number of days taken from positive to negative tests between patients who presented with symptoms and those without symptoms. This is similar to findings by AlBahrani et al. where they found no difference in symptoms among the different Ct values of admitted patients.^22^

Similarly, we found that there was no statistically significant difference in the median time from positive to negative by severity of disease among those who had symptoms. Patients with severe disease did not take longer to shed off the Ct value as predicted.

The duration of viral shedding depends on the type of haematological malignancy, treatment history and severity of COVID-19 disease. Hospitalised patients with COVID-19, hypogammaglobulinemia, recent chemotherapy – especially with Rituximab, and lymphoma, have a higher risk of viral persistence.^28^ These factors impair B-cell response and the production of antibodies with neutralising activity against the virus. One study showed that two patients without peripheral SARS-CoV-2 specific T cells were noted to have had prolonged virus RNA detection even after symptom resolution.^28^ The lack of T-cell responses demonstrates once again, how patients with haematological malignancies fail to mount a protective T-cell response.

As shown by numerous literature reviews and as previously discussed, we also noted that patients with lymphoid malignancies took longer to shed their virus and therefore took longer to switch from a positive to negative test.

Recent studies have reported little to no negative impact of chemotherapy on outcomes of COVID-19 in patients with haematologic malignancies. There was no association between active treatment with poor outcome, including anti-CD20+ treatment. In patients with lymphoid malignancies, despite the anticipated risk of depleting the B-cell compartment and inhibiting humoral immunity, there was also no correlation with a poor outcomes.^29,30,31^

### Limitations

Our study had several limitations beyond its retrospective design. The sample size was relatively small and some data were not retrievable. Additionally, the study was conducted at a single centre, specifically a tertiary hospital, which may introduce a bias towards patients with severe and complicated diseases. Cycle threshold value results are dependent on pre-analytical, analytical and post-analytical factors. These include technique in acquiring the sample (user dependent), sample adequacy, transporting and preservation of the sample, manufacturers’ assay kit and variants detection. Cycle threshold values are also dependent on the time the sample was taken during the infection as they are highest at presentation and decline over days, with longer persistence in immune compromised patients. We had no formal documentation or data on when the NP swabs were done in relation to the onset of symptoms. Some COVID-19 symptoms were similar to that of the underlying haematological malignancy such as fever and anaemia-related dyspnoea and thus being a cofounder. Repeat testing was not well timed or standardised, and often done based on when the patient would be receiving their subsequent inpatient chemotherapy. Because of travel restrictions, changes in access to healthcare facilities and anxiety relating to the pandemic, some patients were lost to follow-up for several weeks and months or having their chemotherapy delayed. This leads to a long gap between repeat swabs, and therefore negatively impacted on the time taken from positive to negative tests. Lastly, the study was conducted during the first two waves of COVID-19 in South Africa, and it is not clear if the same associations would be true for breakthrough infections or reinfections in the current context.

## Conclusion

Our study did not find any association between the initial or presenting SARS-CoV-2 Ct values and clinical symptoms, but showed an association between lower Ct values and severe disease. Viral shedding is prolonged in patients with haematological malignancies and is dependent on multiple factors. Further studies are needed to elucidate the dynamics of viral Ct values and the pathogenesis of the disease in order to understand the disease presentation, progression and outcome. Although there could be some benefit in delaying chemotherapy as patients might have an acute COVID-19 infection with higher Ct values and are therefore at risk for adverse outcomes with early chemotherapy initiation, patient treatment should be individualised and adhere to local infectious disease protocols during seasonal COVID-19 outbreaks.
